# TRPM5-expressing microvillous cells in the main olfactory epithelium

**DOI:** 10.1186/1471-2202-9-114

**Published:** 2008-11-24

**Authors:** Weihong Lin, Ejiofor AD Ezekwe, Zhen Zhao, Emily R Liman, Diego Restrepo

**Affiliations:** 1 Department of Biological Sciences, University of Maryland, Baltimore County, Baltimore, MD 21250, USA; 2 Neuroscience Graduate Program and Department of Biological Sciences, University of Southern California, Los Angeles, California 90089, USA; 3 Department of Cell and Developmental Biology, the Neuroscience Program, and Rocky Mountain Taste and Smell Center, University of Colorado Denver Anschutz Medical Campus, Aurora, CO 80045, USA

## Abstract

**Background:**

The main olfactory epithelium (MOE) in the nasal cavity detects a variety of air borne molecules that provide information regarding the presence of food, predators and other relevant social and environmental factors. Within the epithelium are ciliated sensory neurons, supporting cells, basal cells and microvillous cells, each of which is distinct in morphology and function. Arguably, the least understood, are the microvillous cells, a population of cells that are small in number and whose function is not known. We previously found that in a mouse strain in which the TRPM5 promoter drives expression of the green fluorescent protein (GFP), a population of ciliated olfactory sensory neurons (OSNs), as well as a population of cells displaying microvilli-like structures is labeled. Here we examined the morphology and immunocytochemical properties of these microvillous-like cells using immunocytochemical methods.

**Results:**

We show that the GFP-positive microvillous cells were morphologically diversified and scattered throughout the entire MOE. These cells immunoreacted to an antibody against TRPM5, confirming the expression of this ion channel in these cells. In addition, they showed a Ca^2+^-activated non-selective cation current in electrophysiological recordings. They did not immunoreact to antibodies that label cell markers and elements of the transduction pathways from olfactory sensory neurons and solitary chemosensory cells of the nasal cavity. Further, the TRPM5-expressing cells did not display axon-like processes and were not labeled with a neuronal marker nor did trigeminal peptidergic nerve fibers innervate these cells.

**Conclusion:**

We provide morphological and immunocytochemical characterization of the TRPM5-expressing microvillous cells in the main olfactory epithelium. Our data demonstrate that these cells are non-neuronal and in terms of chemosensory transduction do not resemble the TRPM5-expressing olfactory sensory neurons and nasal solitary chemosensory cells.

## Background

The peripheral olfactory epithelium in mammal is made up of four types of cells, ciliated olfactory sensory neurons (OSNs), basal cells, supporting cells and microvillious cells, which together form a pseudostratified epithelium [1-3]. The olfactory sensory neurons are specialized in detecting diverse odor molecules and transmitting information to the olfactory bulb through their axonal projections [4-6]. Mature OSNs are ciliated bipolar neurons, with cell bodies that are located in the middle layers of the epithelium[1,7-9]. Each of the mature OSNs sends an apical dendrite to the luminal surface where the dendrite terminates in an oval structure, the olfactory knob, bearing approximately 20 cilia where olfactory receptor proteins and the elements of the olfactory transduction cascade are localized [10-12]. A single axon protruding from the basal end of the soma of the OSN penetrates the basal lamina and projects to the olfactory bulb[12].

The supporting cells and basal cells are not sensory cells. The supporting cells also called sustentacular cells are columnar in shape. Their cell bodies span the entire basal to apical extent of the epithelium. Their apical end is covered with long or short microvilli and their somata are located in the superficial layer of the epithelium[2,13-15]. Sustentacular cells are thought to serve a supporting role akin to that of glial cells in the brain. The basal cells, including both globose and horizontal cells, reside basally just above the basal lamina. Amongst these there are olfactory stem cells, capable of regenerating other types of cells in the epithelium throughout life [16-18]. Thus, OSNs, supporting cells and basal cells are distinct in morphology and function.

In contrast, the function of microvillous cells of the mammalian OE is not well understood and they appear to be morphologically diverse. Electron microscopic studies have revealed that their apical microvilli can take different shapes, lengths, and diameters while their cytoplasm can be either electro-lucent or opaque[14,19-22]. Some microvillar cells have been reported to project thin axons to the olfactory bulb, suggesting a second class of bipolar sensory neurons in the olfactory mucosa[1,20,22]. However, the presence of axonal processes is questioned in other studies where surveys with epithelial markers indicate that at least some microvillar cells in the OE are not of neuronal origin and do not bear axonal processes[21,23]. One possibility emerging from these studies is that there are several different types of microvillar cells, some neuronal and others epithelial in nature.

Arguably the best characterized microvillous cells are those found in the OE of fish where they act as sensory receptors that respond to water-borne odors[24,25]. Recent studies have indicated that some microvillous cells in the olfactory epithelium of mammals are chemosensitive as well. Elsaesser et al [26]reported that a subset of microvillar cells express the transient receptor potential channel C6 and other elements of a phosphatidyl-inositide signaling pathway. These cells respond to odorants and appear to have thin axon-like processes although it is not clear whether they reach the olfactory bulb[27]. More recently, we[28] and others [29]provided evidence that TRPM5, an ion channel that is essentially for chemosensory transduction in taste cells [30-32], may be expressed in microvillar cells of the olfactory epithelium, suggesting that these cells are chemosensory. However, it is not yet known whether the TRPM5-expressing cells in the olfactory epithelium resemble OSNs[5], solitary chemosensory cells [33-35] or taste receptor cells[30,31,36] in chemical sensing.

In a previous study we reported that in transgenic mice where the TRPM5 promoter drives the expression of GFP (TRPM5-GFP) a subset of short cells that appeared to be microvillar cells are GFP positive[28]. Here we use immunohistochemical methods to confirm the expression of TRPM5. We also utilize immunohistochemical methods to survey the expression of elements of the sensory transduction pathways in these microvillous-like cells. We find no expression of key transduction elements in these cells that resemble other TRPM5-expressing chemosensory cells. In a companion paper Hansen and Finger utilize electron microscopy to show that these short TRPM5-expressing cells that we call microvillous-like are indeed microvillar cells. Preliminary data has been presented in abstract forms.

## Results

### GFP expression in microvillous-like cells in olfactory epithelia of TRPM5-GFP mice

**Figure 1:**
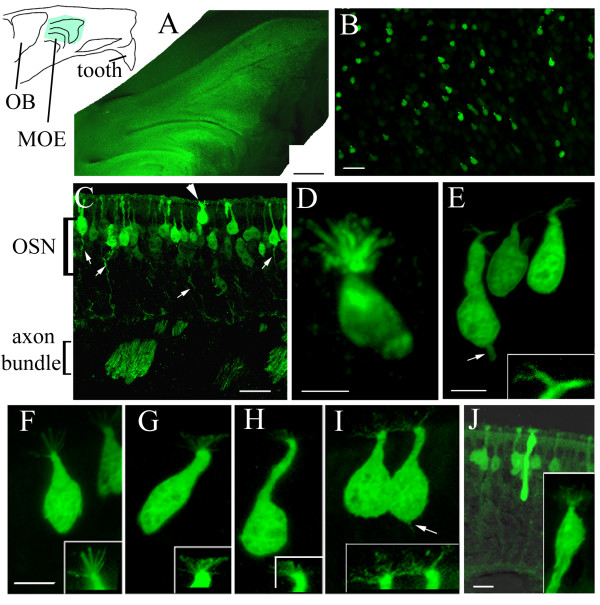
**TRPM5 promoter-driven GFP expression in microvillous cells of the olfactory epithelium. **Top left inset: a draw of a hemi-nose. The blue area corresponds to the image of A. OB: olfactory bulb. A. An epi-fluorescence image from a hemi-nose preparation showing GFP expression in the MOE. B. A confocal image from an endo-turbinate epithelial strip showing that the GFP-expressing cells were scattered and had a pear- or flask-like shape. C. The GFP-expressing cells lie in the most superficial layer of the MOE. Arrow head points to a microvillous cell. In contrast, the GFP-expressing OSNs show apical dendrites and axons (arrows) that form olfactory nerve bundles. D-I. Representative images of GFP-expressing microvillous cells showing various cell shapes and apical processes.  Insets showed apical microvilli with heightened intensity, which could be wavy (D), branched (E), stiff and spreading (F and G), short brush-like (H), or relatively fine (I). These cells did not have axons although some cells showed short basal processes (arrows in E and I). J. A rare type of microvillous cells with a relatively thick and longer basal process extending half of the epithelium. Scales: A, 500 μm; B, C and J, 20 μm; D to I, 5 μm.

Previously we reported on two distinct populations of cells in the olfactory epithelium that were GFP-positive in transgenic mice where the TRPM5 promoter drove the expression of GFP[28]. One population shows morphological and immunocytochemical characteristics of bipolar OSNs that reside preferentially in the lateral and ventral regions of the epithelium. In contrast, the other population of the GFP-expressing cells, which in general displayed stronger GFP expression, was found throughout the OE without apparent zonal segregation. Fig. 1A shows an epi-fluorescence image taken of a whole mount preparation of the medial endo-turbinate of a TRPM5-GFP mouse. Because there are very few TRPM5-expressing OSNs in the medial surface of this region and because the GFP expression in the OSNs is weaker, individual GFP-expressing cells shown in the picture belong to the population of microvillous cells. A higher magnification photomicrograph is shown in Fig. 1B. On average, there were 1158 ± 205 per mm^2 ^GFP-expressing microvillous-like cells in the OE (n = 3). Interestingly, these cells were largely confined to the sensory epithelium, and there were few such cells in the adjacent respiratory epithelium. This is in striking contrast to a distinct population of microvillar cells confined to the respiratory epithelium of the anterior nasal cavity (solitary chemosensory cells) that are thought to detect irritants[33,34,37].

Examination of microscopic images from epithelial sections revealed morphological features of the GFP-expressing microvillous-like cells. First, they were usually about 20 μm in length, shorter than OSNs and supporting cells and their shape varied from cell to cell resembling a flask, a bottle or a pear. Their somata were located in the superficial (apical) layer of the pseudostratified epithelium where the nuclei of supporting cells are located (Fig. 1C, arrowhead). Second, most of these GFP-expressing cells did not show dendritic knobs and cilia typical for the bipolar OSNs. Instead these cells showed microvilli-like processes in the apexes that displayed diverse morphology under light microscopy. In some experiments we used an anti-GFP antibody to intensify the GFP signal. Out of a total 82 cells scanned in a confocal microscope or examined, we found 58% cells showed relatively wavy processes (Fig. 1D), some with relatively fine apical processes (Fig. 1I). Eleven percent of the cells displayed a few thick processes that branched at their distal ends (Fig. 1E and inset for apical processes). Seventeen percent of the cells showed spreading and stiff apical processes, which could be straight and curved at their distal ends (Fig. 1F, G and insets). Twelve percent of the cells showed relatively short and stiff microvilli (Fig. 1H). Hansen and Finger (article in this issue) examined the apical processes using immunolabeling of a microvillus marker espin and electron microscopy. Their results show indeed GFP-expressing microvillous-like cells bear microvilli. In rare case (seven out of 2990 cells examined) we found spindle-like cells that had relatively large diameter basal processes. These cells usually displayed a cluster of apical weaving processes and their somata were located in the same superficial layer as the other short GFP cells described above (Fig. 1J). Third, unlike OSNs that displayed a thin axonal process extended below the basal lamina (Fig. 1C), the TRPM5-expressing microvillous cells did not show axonal processes. In 2990 cells examined in coronal sections from three animals, none showed axons extending to the basal lamina, although about seven percent of the cells showed short basal process (arrow in Fig. 1E). Some cells only showed a potential initiation site of a basal process under the light microcopy (arrow in Fig. 1I). The GFP positive microvillous cells thus are different from the microvillar cells that show slender axonal processes (Fig. 1C) [26]. The lack of an axonal process was confirmed by electron microscopy (Hansen and Finger in this issue).

### GFP-expressing microvillous cells are immunoreactive for TRPM5 and have a Ca^2+^-activated cation current

**Figure 2:**
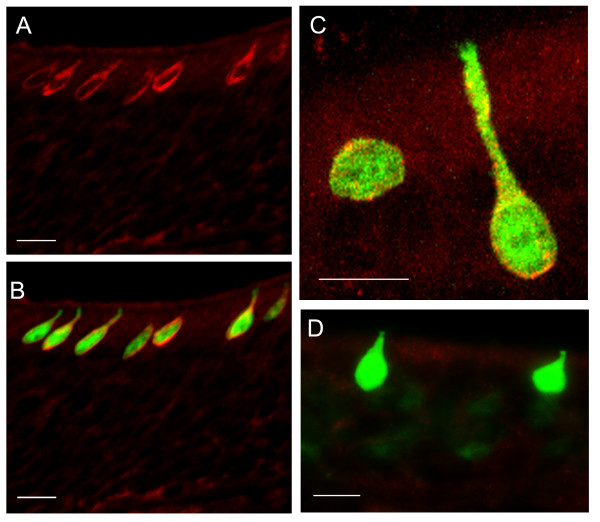
**Immunoreactivity of TRPM5 in GFP-expressing microvillous cells.** A. A confocal image showing that positive immunoreaction for TRPM5 (red) was present in cells located superficially. B. The image of GFP was overlaid onto A, showing co-localization of TRPM5 immunoreactivity and GFP expression. The images were acquired from a section of medial surface of the endo-turbinate where there were no TRPM5-expressing ciliated OSNs. C. A magnified image showing the TRPM5 immunoreactivity (red) in GFP expressing cells. D. Negative control. The antibody did not label GFP expressing cells in the olfactory epithelia of TRPM5-KO-GFP mice. Scales: 20 μm.

TRPM5 forms Ca^2+^-activated cation channels in heterologous cells and taste receptors cells and is essential for normal taste sensation[30,31,38-42]. Based on these results, it is likely that TRPM5 transduces a PLC signaling cascade, initiated by binding of tastants to G protein-coupled receptors, into an electrical response in taste cells. To determine whether TRPM5 is also expressed in microvillous cells in the OE, where it might have a similar function, we performed immunolabeling of sections of MOE with an antibody against the TRPM5[42]. The antibody labeled all the GFP-positive microvillous cells (Fig. 2) in a pattern suggestive of plasma membrane expression of the TRPM5 channel. Controls consisted of the lack of fluorescence upon omission of the primary antibody or in MOE tissue sections from the olfactory epithelium of mice defective for TRPM5 [41](Fig. 2D). These results demonstrate that TRPM5, an ion channel known to be involved in chemical sensing was expressed in these microvillous cells.

**Figure 3:**
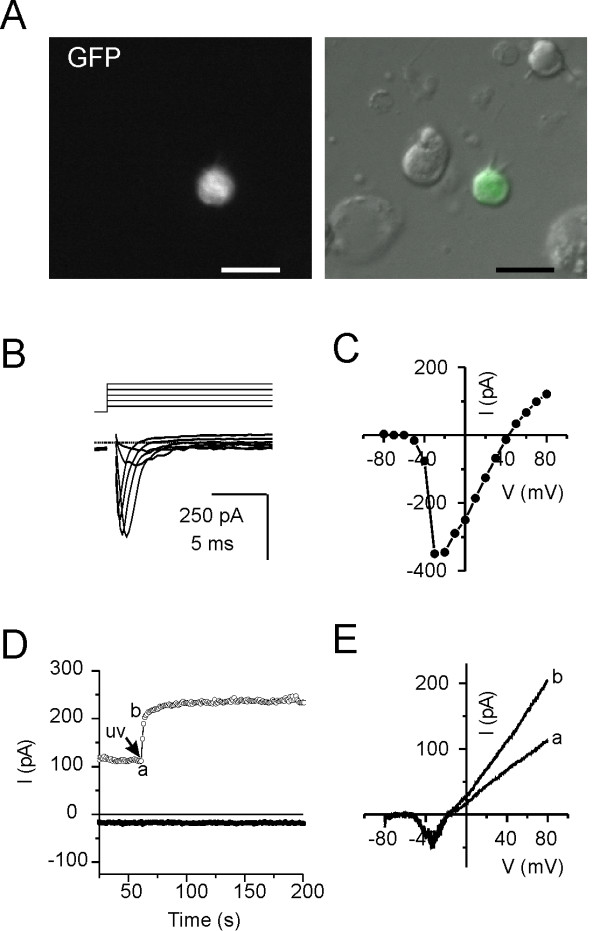
**GFP positive microvillous cells from mouse olfactory epithelium have voltage-gated Na^+ ^currents and a Ca^2+^-activated cation current.** A. Isolated cells from mouse olfactory epithelium used for patch clamp recording were identified based on GFP fluorescence and the presence of a microvillous structure. Scale bar is 10 μm. B. Currents in response to voltage steps from -80 mV to from -50-0 mV, in 10 mV increments. Dotted line indicates the zero current level. Note the appearance of a fast voltage-activated Na^+ ^current in this cells. C. Peak inward current as a function of voltage from the experiment in (A). D. Current in response to UV uncaging of DMNP-EDTA (caged Ca^2+^). Note the large increase in the outward current (measured at +80 mV; open symbol) and absence of response at -80 mV (filled symbol). E. Currents in response to ramp depolarization at time (a) and (b) from the experiment shown in D. Data are representative of three independent experiments.

To determine whether TRPM5 channels in microvillous cells are functional, we recorded from freshly dissociated GFP-labeled cells isolated from the olfactory epithelium of TRPM5-GFP transgenic mice. Cells were further chosen based on the presence of a stubby dendrite and obvious microvillar processes (Fig. 3A). Note that in this preparation from septal olfactory epithelium, no GFP-positive ciliated OSNs were observed. With intracellular Cs^+ ^in the pipette, step depolarizations revealed the presence of a voltage-activated Na^+ ^current which activated at -50 mV (Fig. 3B, C). This current is also evident in recordings of responses to ramp depolarizations (Fig. 3E). UV uncaging of Ca^2+ ^activated a current of ~100 pA at +80 mV (Fig. 3D, E). This current was strongly outwardly rectifying (compare outward and inward currents in Fig. 3E at times a and b), and therefore cannot be attributed to a change in the leak current across the patch. Interestingly, while the TRPM5-current recorded from mouse taste receptors cells was found to decay following activation by uv uncaging of Ca^2+^[42], this current was sustained. This suggests a possible difference in the inactivation of the channel in different cell types. The small magnitude of the current was consistent among all cells studied (n = 3) and could not be attributed to the UV uncaging protocol, which in control experiments generated a large response in HEK cells transfected with mTRPM5 (not shown). Thus a Ca^2+^-activated cation current is present in these microvillous cells.

### GFP-expressing microvillous cells do not express olfactory signaling elements and marker proteins

**Figure 4:**
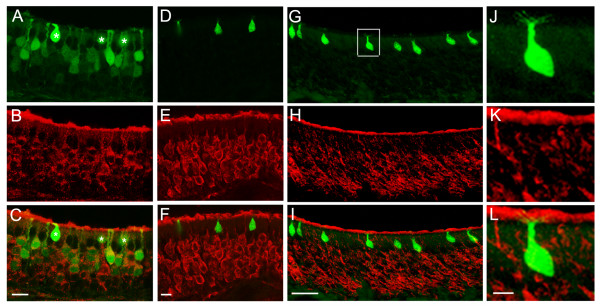
**Negative immunoreactivity for CNGA2, OMP, and a neuronal marker PGP 9.5 in the TRPM5 (GFP)-expressing microvillous cells.** Sections were immunoreacted to antibodies against the CNGA2 (A-C), OMP (D-F), and PGP 9.5 (G-L) respectively. A, D, and G. Fluorescence images showing GFP-expressing microvillous cells in the OE. The microvillous cells in (A) are marked with asterisks to distinguish from the GFP-expressing OSNs. B, E, and H. Fluorescence images of immunoreaction to antibodies against the CNGA2 (B), OMP (E) and PGP 9.5 (H) respectively. All three antibodies labeled OSNs, However, these antibodies failed to label any TRPM5 (GFP)-expressing microvillous cells. C, F, and I. Overlaid images showing there was no co-localization of GFP signal and OMP- or CNGA2- or PGP 9.5 immunoreactivity in microvillous cells. Note in (C) where both GFP-positive OSNs and microvillous cells are present, GFP-expressing OSNs showed co-localization of CNGA2 immunoreaction. J, K, and L. Enlarged images of G-L showing the anti-PGP9.5 antibody did not label a GFP-positive microvillous cell. There were also apparently no PGP9.5-labeled nerve fibers that closely associated to the cell. Scales: A-F, 10 μm; G-I, 20 μm; J-L, 5 μm.

We examined the expression of OSN markers in the TRPM5-expressing microvillous cells using immunolabeling. Most OSNs express the cyclic nucleotide-gated channel subunit A2 (CNGA2), an ion channel subunit essential for odor-induced depolarization of OSNs through the canonical cAMP pathway[5]. As shown in Fig. 4B and consistent with a previous report[43], the anti-CNGA2 antibody labeled the majority of OSNs. This antibody did not label the GFP-positive microvillous cells (Fig. 4A–C). In addition, there is a small subset of OSNs that do not express the CNGA2 and uses a different transduction pathway. These OSNs can be identified by PDE2A immunoreactivity[44]. The anti-PDE2A antibody also failed to label any TRPM5-expressing microvillous-like cells (data not shown). Further, an antibody against the olfactory marker protein (OMP), a marker for mature OSNs that labeled the TRPM5 expressing OSNs[28], failed to label microvillous-like cells (Figs. 4D–F). The absence of labeling of microvillar cells by the OMP antibody is consistent with a previous report[45]. Thus, the TRPM5-expressing microvillous cells do not express common olfactory transduction elements and olfactory marker protein.

### Lack of immunolabeling of microvillous cells by the pan-neuronal marker PGP9.5

The negative immunolabeling of CNGA2 and OMP in microvillous cells raise the question of whether TRPM5-expressing microvillous cells are neurons. We tested this by immunolabeling sections of the olfactory epithelia with PGP9.5, a marker expressed by all neurons including OSNs[46]. As shown in Fig. 4G–I the PGP9.5 antibody labeled all the OSNs strongly, but did not label the short TRPM5-expressing cells. The enlarged images are shown in Fig. 4J–L, suggesting that these microvillous cells are likely not neurons.

### Lack of immunolabeling with markers that label solitary chemosensory cells

**Figure 5:**
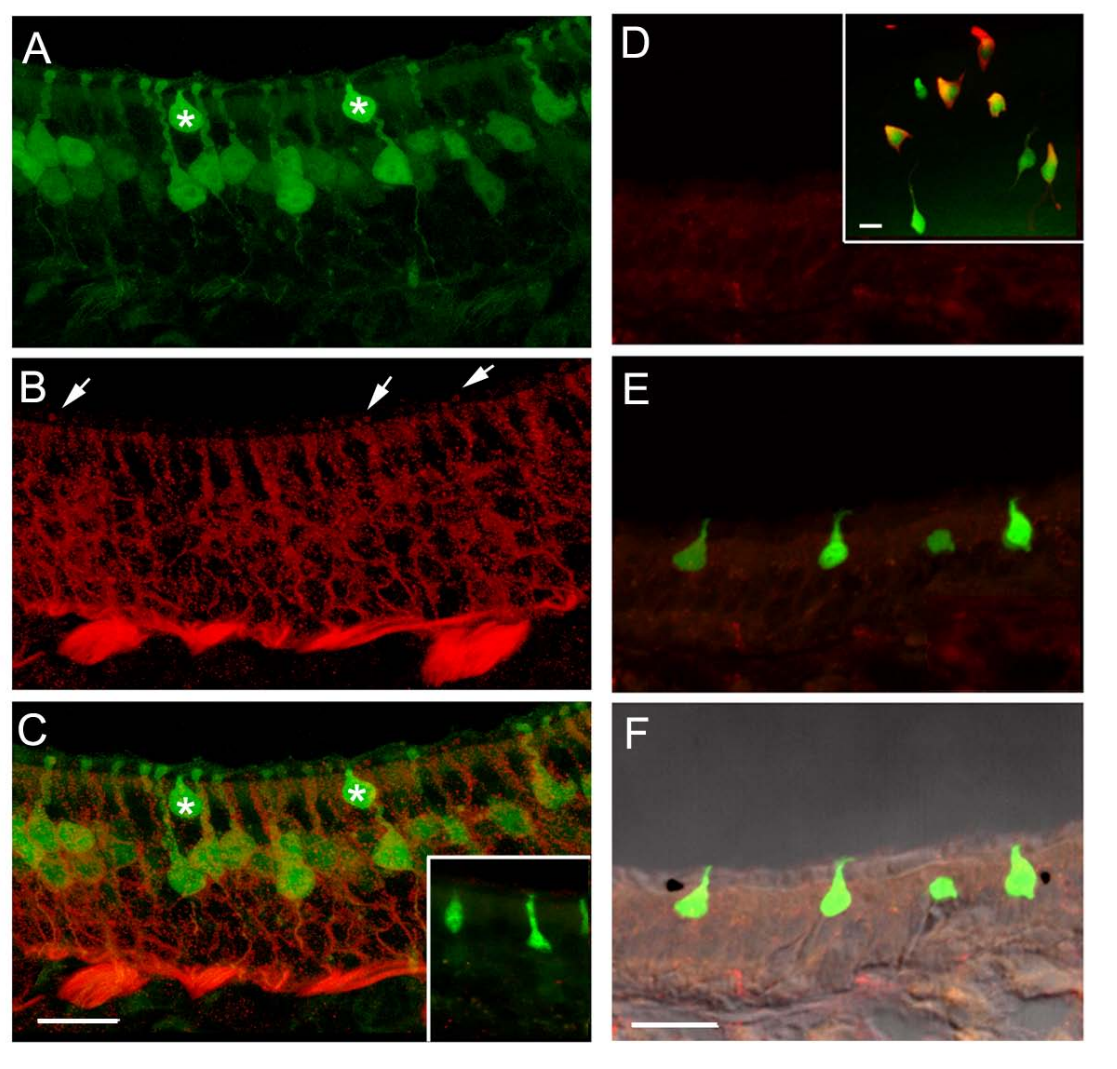
**Negative immunoreactivity for PLCβ2 and α-gustducin in the TRPM5-expressing microvillous cells.** A. An image of GFP-expressing microvillous cells (asterisks) and OSNs. B. The antibody against the PLCβ2 labeled OSNs in a pattern of the membrane of OSNs and some apical regions pointed by arrows, which might be apical microvilli of a different population microvillous cells reported previously[26]. C. Overlaid image of A and B showing the antibody did not label the TRPM5-expressing microvillous cells. Inset is an image from a control experiment, in which the PLCβ2 antibody was pre-absorbed to the specific antigen peptide, showing no immunoreactivity. D. The antibody against α-gustducin did not label the TRPM5-expressing microvillous cells in the OE, although the antibody at the same condition labeled nicely the TRPM5-expressing solitary chemosensory cells in the respiratory epithelium (inset). E. Image of the TRPM5-expressing microvillous cells from the same OE section overlaid with the image of D, showing there was no α-gustducin immunoreactivity in these microvillous cells. F. The image of E was overlaid with the bright field image. Note the apical microvilli of these cells reach to the lumen. Scales: 20 μm; inset, 10 μm.

Solitary chemosensory cells in the respiratory epithelium at the anterior end of the nasal cavity express TRPM5[29,33,37]. In addition many solitary chemosensory cells also express the G-protein α subunit gustducin and the enzyme phospholipase C β2 (PLC β2)[33,34], both of which are implicated in chemical sensing by taste cells[31,36,47]. PLCβ2 is also present in bipolar microvillar cells that respond to odorants[27]. Fig. 5A shows a section of the MOE, where GFP is expressed in both OSNs and microvillous cells (marked by asterisks). As shown in Fig. 5B–C, the antibody against PLCβ2 labeled the OSNs, but failed to label any microvillous cells. Pre-absorbing the antibody with the antigen peptide abolished the immuno-reaction in a control experiment, demonstrating the specificity of the antibody (Fig. 5C, inset). Negative immunoreaction of the antibody against α-gustducin was also observed in TRPM5-expressing microvillous cells (Fig. 5D–F) at concentration and methods that labeled solitary chemosensory cells in parallel experiments (inset in Fig. 5D). We conducted antigen retrieval and increased the concentration of antibodies but none of the treatments produced positive results. Thus, our data indicate that the immunocytochemical properties of TRPM5-expressing microvillous cells were different from those of solitary chemosensory cells in the respiratory epithelium.

### Lack of peptidergic trigeminal innervation of microvillous cells

**Figure 6:**
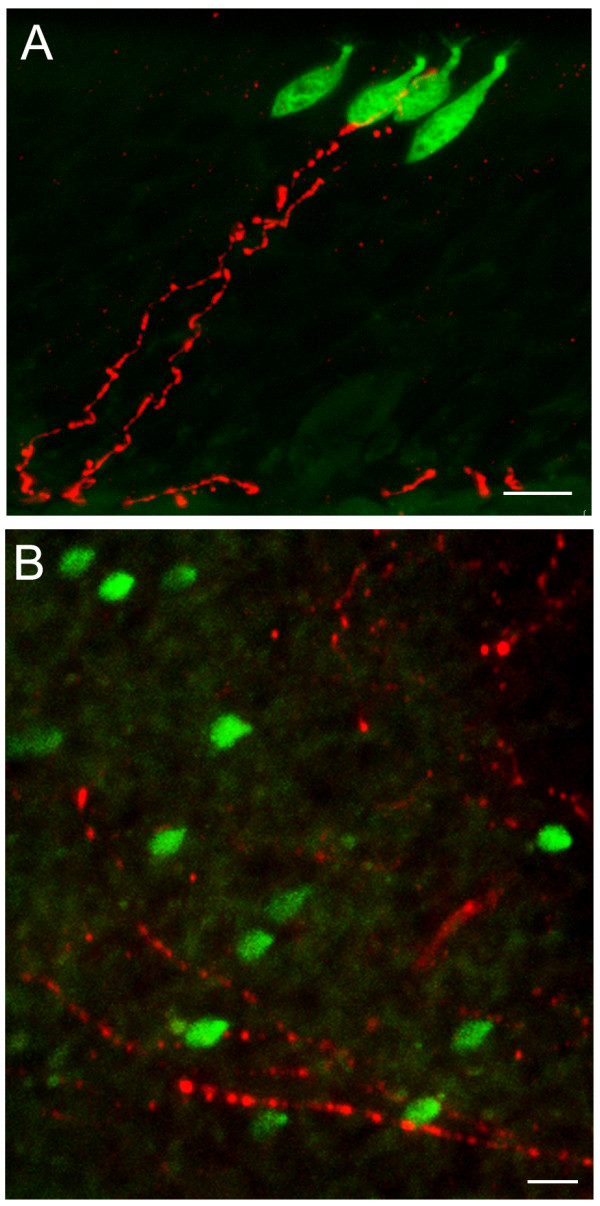
**Immunolabeling of trigeminal peptidergic fibers in the OE.** A. The antibody against the peptide CGRP labeled intraepithelial trigeminal fibers in the OE, Some fibers reached to the superficial layer where the TRPM5-expressing microvillous cells were located but was found adjacent only to a small subset of cells, and did not wrap tightly around the cell bodies as seen for TRPM5-expressing solitary chemoreceptor cells of the anterior nasal epithelium[33]. B. Image from an epithelial strip reacted with the antibody against substance P. Most of the substance P positive fibers ran at the base of the epithelium, with some intraepithelial fibers. Note that most of the GFP expressing microvillous cells did not associate closely with the fibers. Scales: 10 μm.

Solitary chemosensory cells in the respiratory epithelium are innervated by trigeminal peptidergic fibers [33,34,37]. Since there was no apparent axonal projection from the TRPM5-expressing microvillous cells, we investigated whether these cells receive trigeminal innvervation. Epithelial sections and strips were immunoreacted with antibodies against CGRP and substance P, two markers for the trigeminal peptidergic fibers. Most of the labeled nerve fibers were found in the basal lamina of the OE, with some processes traversing the epithelium proper. We found very few CGRP- (Fig. 6A) or substance P- (Fig. 6B) positive fibers close to the TRPM5-expressing microvillous cells. Most of the cells did not associate with immunoreactive fibers. Thus, in contrast with the innervated chemosensory cells in the respiratory epithelium, the microvillous cells display sporadic association with trigeminal fibers making it is unlikely that trigeminal peptidergic fibers carry signals from these microvillous cells. This observation is consistent with the results in Fig. 4J–L, where there is also no closely associated PGP 9.5-expressing nerve fibers coursing along or wrapping the GFP-positive microvillous cells, suggesting lack of nerve innervation of these cells in general.

## Discussion

Our results provide morphological and immunocytochemical evidence that a non-olfactory neuronal type of TRPM5-expressing microvillous cells are present in the mouse olfactory epithelium. These TRPM5-expressing cells are located preferentially in the superficial layer and are scattered throughout the entire OE. Predominant morphological features include diversified apical microvilli and absence of an apparent axon. We confirmed the TRPM5 expression using transgenic mice and immunolabeling methods. Further, we showed that the TRPM5-expressing microvillous cells did not express the transduction components and markers of OSNS, CNGA2, OMP and PGP9.5, nor did they express PLCβ2 or α-gustducin, which are present in many solitary chemosensory cells of the respiratory epithelium. Therefore, the microvillous TRPM5-expressing cells belong to a unique population of cells, different from other TRPM5-expressing OSNs and solitary chemosensory cells in morphology and in the expression of cell markers. The fact that TRPM5-expressing microvillous cells do not express elements of the transduction pathways from other chemoreceptors, do not have axonal processes evident under light microscopy (and under electron microscopy see accompanying article by Hansen and Finger) and are not innervated by the trigeminal peptidergic fibers calls into question the role of these TRPM5-expressing cells as chemoreceptors. Alternately, there is a possibility that these microvillous cells detect chemical stimuli and transmit their signal to surrounding cells (sustentacular and/or olfactory sensory neurons) through non-synaptic transmission. This would be analogous to type II taste cells that detect bitter and sweet compounds and transmit the signal through non-vesicular release of ATP[48]. If this is the case, transduction would have to be mediated by elements that we have not surveyed in this study.

### Microvillous cells in the olfactory epithelium

Microvillous cells have been discovered in the OE in a number of species[25,49-53]. Ultrastructural analyses have shown that the apical processes of microvillous cells can be rigid; finger-like; aligned in parallel with a more uniform diameter and length; more compacted forming a cone shape. Some microvilli reportedly are shorter resembling the brush cells in the respiratory epithelium, while some others resemble the hair cells in the cochlea[54]. Our study further demonstrates the diversity of apical processes (Fig. 1). Although it is not known whether the diversity of apical microvilli indicate functional specializations, expression of TRPM5 suggests that these cells may utilize a common signaling pathway.

In some microvillous cells, single slender axon-like processes that protrude from the basal regions of individual cells are clearly visible[20,26]. When the cytochemical tracer macromolecule horseradish peroxidase is injected to the olfactory bulb, some microvillous cells are backfilled. The result demonstrates axonal projection and suggests that as found in fish, there is a second type of olfactory sensory neuron bearing microvilli, with distinct a morphology[22]. In contrast, some microvillar cells do not show axons-like processes[21]. Immunolabeling with epithelial markers suggest that at least some microvillous cells are non-neuronal[23,51]. However, the microvillar cells described by Elsaesser et al [27] differ from the epithelial type microvillar cells in morphology, particularly the basal processes. Our study further documented the difference between the microvillous cells expressing the PLCβ2 signaling pathway [27]and TRPM5-expressing cells. In our study, the TRPM5-expressing microvillous cells did not express PLCβ2. Further, antibodies against PGP 9.5, OMP and CNGA2 that labeled OSNs, all failed to label the TRPM5-expressing microvillous cells, strongly suggesting that the TRPM-5-expressing cells were not neurons. Thus, our study revealed further diversity of the microvillous cells in the OE.

At present, the physiological function of the epithelial type microvillous cell in rodents is not known, although there is evidence that some microvillous cells in mammals are chemosensory[27]. Since the TRPM5-expressing microvillous cells in mice did not express distinct cell marker and signaling proteins of OSNs and microvillous cells that possess axons, the TRPM5-expressing cells may function distinctly from the OSNs in the OE and other chemosensory neurons. One possibility is that TRPM5, which is temperature sensitive [55], functions as a thermosensor to monitor temperature of inhaled air. Further experiment will be needed to determine the physiological function of the microvillous cells and role of the TRPM5.

### Difference between TRPM5-expressing microvillous cells in the MOE and solitary chemosensory cells

Solitary chemosensory cells are thought to constitute a diffuse chemosensory system in the nasal cavity[35]. These cells were first discovered in aquatic animals[56], later found to be in mammalian nasal respiratory epithelium[29,34,35] as well as epithelia of respiratory and gastrointestinal tracts based on the expression of signaling components such as α-gustducin, PLCβ2 and TRPM5 known to be critical for chemical sensing in taste receptor cells[29,57]. The solitary chemosensory cells in the anterior nasal cavity respond to chemical irritants[33,34,37]. Kaske et al [29] further found that TRPM5 is expressed in brush cells of the epithelia of respiratory and gastrointestinal tracts, The authors consider TRPM5 to be an ubiquitous signaling component in chemosensory cells and TRPM5-expressing microvilli cells to be chemosensory. However, despite the expression of TRPM5, the microvillous cells of the olfactory epithelium do not express α-gustducin and PLCβ2 typically found in the solitary chemosensory cells. It is unlikely that TRPM5-expressing microvillous cells utilize the same PLC signaling mechanism to detect chemicals.

## Conclusion

Our study provides evidence that TRPM5-expressing microvillous cells represent a group of morphologically diversified non-neuronal cells in the olfactory epithelium of mice. These cells are unique among TRPM5-expressing microvillous cells in that they do not express sensory signaling elements typically found in TRPM5-expressing olfactory neurons and chemonsensory cells. Further studies are needed to determine physiological function of the TRPM5-expressing microvillous cells.

## Methods

### Animals

Adult C57BL/6 background TRPM5 transgenic mice were used (kindly provided by Dr. Robert R Margolskee) [58]. Offspring were genotyped using the polymerase chain reaction (PCR) for the presence of GFP. All animal care and procedures were in compliance with the Animal Care and Use Committees of University of Colorado Denver Anschutz Medical Campus, University of Maryland, Baltimore County, and University of Southern California.

### Immunocytochemistry

#### Tissue preparation

Mice were anesthetized with ketamine/xylazine (100 μg-20 μg/g body weight) or tribromoethanol (Avertin 250 μg/g body weight), perfused transcardially with 0.1 M phosphate buffer (PB) followed by a PB buffered fixative containing 3% paraformaldehyde, 0.019 M L-lysine monohydrochloride, and 0.23% sodium m-periodate[59]. The nose was harvested and post-fixed for 2 h. For immunolabeling using tissue sections, bones surrounding the nose were removed after fixation and tissues were transferred into 0.1 M phosphate buffer saline (PBS) with 25% sucrose overnight and embedded with embedding optimal cutting temperature (OCT) compound (Sakura finetek USA Inc, Torrance CA). Fourteen micron transverse sections were cut using a Micron cryostat, mounted onto Superfrost plus slides (Fisher Sci, Pittsburgh, PA) and stored in -80°C freezer until used.

#### Immunocytochemistry

Sections containing MOE or stripped olfactory epithelium were rinsed in 0.1 M PBS and incubated in PBS buffered blocking solution containing 2% normal donkey serum, 0.3% Triton X-100 and 1% bovine serum albumin for 1.5 hour. Sections were then incubated overnight or 72 hours with primary antibodies against each of the following proteins: TRPM5 (1:500; [42], PLCβ2 (1:200; Cat No: sc-206, Santa Cruz Biotechnology, Santa Cruz, CA), calcitonin gene related peptide (CGRP) (1:500, Cat# IHC6006, Peninsula Lab. San Carlos, CA), Substance P (1:1000, Cat No: AB1977, Chemicon, Temecula, CA), α-gustducin (1:1000, Cat No: sc-395, Santa Cruz Biotechnology, Santa Cruz, CA) or PGP9.5 (ubiquitin carboxyl-terminal hydrolase; 1:500, Cat No: 7863-0504, Biogenesis, Sandown, NH), OMP (1:6000, kindly provided by Dr. Frank Margolis, from the University of Maryland, School of Medicine), CNGA2 (1:200, Alamone, Labs, Ltd, Jerusalem, Israel). For some experiments we also used anti-GFP (1:2000, Cat No:AB16901, Chemicon) to intensify the GFP signals. After incubation of the primary antibodies, sections were then washed and reacted with donkey anti-rabbit secondary antibody (Alexa 555, Probes, Eugene, OR) or donkey anti-Chicken secondary antibody (Jackson ImmunoResearch, West grove PA) for 1 hour at room temperature. Sections were mounted on slides with Fluoromount-G (SouthernBiotech, Birmingham, AL). Controls for these experiments consisted of removing primary antibodies, pre-absorbing the antibodies with specific antigen peptides (1:10 ratio of antibody to antigen), and using tissues from the TRPM5KO mice for the anti-TRPM5 antibody, all resulting in negative labeling.

#### Image acquisition

For direct visualization of GFP expression, the nose was split along the midline to expose the nasal cavity. Low magnification pictures were taken using an Olympus BX 41 compound microscope equipped with epi-fluorescence. High magnification whole-mount images on stripped epithelia containing GFP-positive cells were taken using an Olympus Fluoview confocal microscope, or a Leica TCS SP5, or an Olympus BX 61 epi-microscope equipped with a spinning disc confocal unit.

#### Cell counting

For estimation of the number of TRPM5 (GFP)-expressing cells in olfactory epithelium, the TRPM5-GFP mice were fixed, and the nose was split along the midline. The olfactory epithelia from three noses were stripped, spread out and mounted onto microscope slides with fluoromount-G. Multiple images at random region were taken at low magnification (4×) using an Olympus epi-fluorescence microscope and the respiratory epithelium from the hemi-noses was reconstructed.

### Patch clamp electrophysiology

Cells in the MOE were isolated as previously described [60]. Following dissociation, cells were allowed to settle on the bottom of an uncoated coverglass, and microvillar cells were identified for recording by the presence of GFP fluorescence and morphology. Bath solution was: 145 mM NaCl, 5 mM KCl, 1 MgCl_2_, 2 mM CaCl_2_, 20 mM Dextrose, 10 mM HEPES (pH 7.4 with NaOH). Internal solution contained: 120 mM CsAsp, 20 mM CsCl, 10 mM HEPES, 2.5 mM 2,6-dimethyl-4-nitropyridine DMNP-EDTA (Invitrogen Molecular probes, Eugene, OR), 0.75 mM CaCl_2_, pH 7.4. CsCl 22; Aspartic acid 111; HEPES 11; MgATP2.2; Na_2_ATP 3.3 (pH 7.2 with CsOH). UV light (100 ms) was delivered from a mercury arc lamp that was then passed through a 350/50× bandpass filter (Chroma Technology Corp, San Diego, CA) controlled by a uniblitz shutter (Vincent Associates, Rochester, NY), as previously described[42]. Patch clamp electrophysiology was performed as previously described[39,60].

## Authors' contributions

WL designed and carried out the majority of the experiments, data analysis, and drafting of this manuscript. EADE, Jr performed some immunolabeling and data collection. ZZ generated the antibody for TRPM5 and performed some of the immunolabeling experiments. ERL supervised the generation of the TRPM5 antibody and the electrophysiological recordings and contributed to the data analysis and writing of the manuscript. DR supervised the study and participated in its design, coordination, editing and completion.
